# The 2 years’ long-term efficacy and safety of peroral endoscopic myotomy for the treatment of achalasia: a systematic review

**DOI:** 10.1186/s13019-018-0811-9

**Published:** 2019-01-03

**Authors:** Huan Li, Wei Peng, Shu Huang, Yutang Ren, Yan Peng, Qing Li, Jiao Wu, Xiangsheng Fu, Xiaowei Tang

**Affiliations:** 1 Department of Gastroenterology, Affliated Hospital of Southwest Medical University, Street Taiping No.25, Region Jiangyang, Luzhou, 646099 Sichuan Province China; 2 Department of Gastroenterology, the People’s Hospital of Lianshui, Huaian, China; 3 Departmemt of Gastroenterology, Beijing Tsinghua Changgung Hospital Medical Center, Tsinghua University, Beijing, China; 4 Department of Gastroenterology, Affiliated Hospital of North Sichuan Medical College, Road Wenhua 63#, Region Shunqing, Sichuan, 637000 China

**Keywords:** Peroral endoscopic myotomy, Achalasia, Clinical success, Complications, Efficacy, Systematic review

## Abstract

**Aim:**

In this retrospective review, we aimed to investigate the long-term efficacy and safety of POEM with follow-up period over 2 years.

**Materials and methods:**

A systematic review related to POEM for achalasia was conducted. A literature search was performed in Pubmed, Medline, Ovid, Cochrane and EBSCO databases on November 2017. The following postoperative outcomes were extracted: Eckardt score, lower esophageal sphincter pressure, complications and clinical success.

**Results:**

The total number of patients was 373. The mean operative time was 66.7 min and the overall rate of complications was 21.2%. The mean follow-up period was 30.0 months. The overall clinical success rate was 92.9% and the rate of gastroesophageal reflux disease was 10.2%. Rate of mortality after POEM was 0.

**Conclusions:**

Our study demonstrated that POEM is effective and safe for treating achalasia during the long-term followed up over 2 years.

## Introduction

Achalasia is characterized by aperistalsis of the esophagus and impaired relaxation of the lower esophageal sphincter [1, 2]. Pathologic mechanisms of achalasia remain unknown, although various studies have reported that virus, inflammation, and autoimmune mechanisms may affect the neuronal degeneration of esophageal ganglion cells [1]. Patients with achalasia have typical clinical manifestations, including dysphagia, retrosternal pain, reflux, heartburn, and weight loss [3–5].

The development of medical technology and equipment has led to a breakthrough in the treatment of achalasia. Initial interventions included drug therapy, endoscopic balloon dilatation (EBD), botulinum toxin injections (BTI), and laparoscopic Heller myotomy (LHM). For nearly 100 years, the surgical approach to achalasia was based on an open Heller procedure. However, over the last 20 years, LHM has become a routinely performed procedure, with obvious advantages compared with the open approach [1]. However, each of these interventions has both advantages and disadvantages. The effectiveness of drug treatment is shorter and the recurrence rate is higher. To ensure its effectiveness, BTI should be used for repeated treatment. Likewise, EBD may require repeated treatments, and LHM often requires an additional fundoplication procedure to reduce the occurrence of gastroesophageal reflux disease [5, 6]. Endoscopic myotomy for achalasia was first reported by Ortega et al. in 1980 [7]. Pasricha et al. reported peroral endoscopic myotomy (POEM) procedures in animal models of pigs in 2007 [8]. Inoue et al. reported the feasibility and effectiveness of POEM in 2010 [9]. Since then, POEM has been widely performed, and several studies have reported its long-term efficacy and safety [10, 12, 13]. Therefore, our systematic review was designed to explore the 2 years’ long-term effectiveness and safety of POEM.

## Methods

### Literature retrieval

The investigation for this systematic review was based on the principle of PRISMA [11]. The search strategy was comprehensive. Electronic database searches were conducted in PubMed, Medline, EBSCO, Cochrane, and Ovid databases during November 2017 using the following search terms: “peroral endoscopic myotomy,” “achalasia,” “POEM,” “laparoscopic Heller myotomy,” “esophageal motility disorder,” and related words. Studies that met the following inclusion criteria were analyzed for pooled analysis: (i) English language literature, (ii) the full text of literature, and (iii) the original article. Excluded criteria were meta-analysis and systematic reviews, animal trials, case reports, non-English language literature, a mean follow-up period of < 24 months, and no reported follow-up period.

### Data extraction

Data were independently extracted by two investigators (H. Li and W. Peng). If one researcher was unable to confirm data, then both researchers conferred about whether to include these data. The following data were abstracted: (i) the country in which original articles were published; (ii) the number of patients and proportion of men and women; (iii) patients’ average course of illness and average age; (iv) the average operation time and mean hospital stay; (v) the mean follow-up time; (vi) the number of patients who had previously received interventions; (vii) the average length of the submucosal tunnel under dissection; (viii) the total length of the muscle incision, including the mean length of the esophagus and stomach; (ix) the number and proportion of successful procedures; (x) the complications, replacement therapy, lower esophageal sphincter pressure (LESP), and Eckardt score; and (xi) the number and proportion of patients with gastroesophageal reflux (GER) disease and reflux esophagitis (RE). To obtain information that might be lost in the literature, we attempted to contact the co-authors of the original articles.

### Definitions

(i) Patients’ symptom duration: the time from the onset of clinical symptoms to medical attention;
(ii) Length of the submucosal tunnel: the length of the dissected tunnel determined by endoscopy, including the esophagus and stomach;
(iii) Intervention measures: previously used treatment for achalasia, for example, drug therapy, EBD, BTI, LHM, and POEM;
(iv) Clinical success: significantly reduced symptoms of achalasia during the follow-up period; postoperative Eckardt score ≤ 3; significantly reduced LESP;
(v) Complications and GER/RE: adverse events that occurred during and after the procedure, including mucosa perforation, esophageal perforation, subcutaneous emphysema, intraoperative/postoperative bleeding, pneumothorax, pneumoperitoneum, atrial fibrillation, urinary retention, pleural effusion, aspiration pneumonia, mediastinal emphysema, and delayed bleeding [6].

### Statistical analysis

Extracted data were summarized using standard calculation principles. Quantitative data were described as mean and ranges or median ± standard deviation and ranges. Categorical variables were expressed as percentages. We used the descriptive statistical method to analyze and conduct our study.

## Results

### Search results

**Fig. 1:**
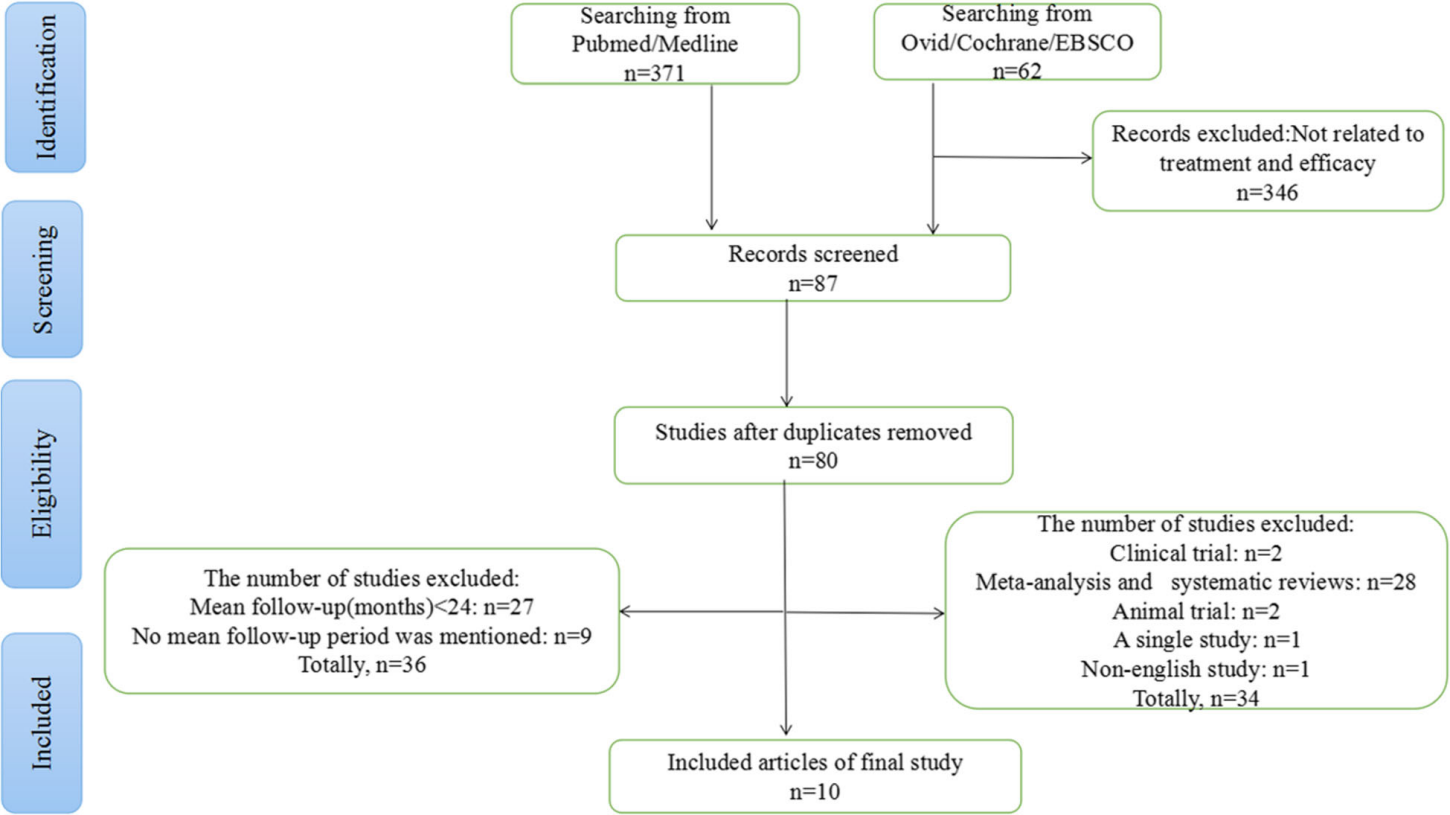
PRISMA flowchart for search strategy of this systematic review

In total, 433 articles were retrieved. Of these, 371 were obtained from PubMed and Medline. In addition, 62 related studies were retrieved from the Ovid, Cochrane, and EBSCO databases. Excluded literature included (i) 346 articles in which treatment and effectiveness were not mentioned; (ii) meta-analysis, systematic reviews, animal experiments, case reports, and non-English language literature (*n* = 34); (iii) follow-up period of < 24 months (*n* = 27); and (iv) the average follow-up period not mentioned (*n* = 9) (Fig. 1). Finally, 10 eligible studies, published between January 2015 and November 2017, were included [12–21].

### Demographic and operational parameters

Demographic characteristics of patients were described in Table 1. Eight studies were from China [12–19] and two were published in the United States [20, 21]. A total of 373 patients (men, *n* = 178; women, *n* = 195) were included in the 10 studies. Patients’ mean age was 42.1 years. The mean symptom duration of all patients was 52.5 months. There were 132 (35.4%) patients who had received other interventions. Previous measures reported included EBD in 33 (8.8%) patients; BTI in 12 (3.2%); LHM in 46 (12.3%); and other treatments in 39 (10.5%) (Table 1). A total of 372 (99.7%) patients successfully underwent POEM. The only one failed procedure was due to serious inflammation and adhesion of the esophagus [15]. The average operational time was 66.7 min, and the mean length of the submucosal tunnel was 11.72 cm. The mean length of total myotomy was 9.3 cm (mean myotomy length in the esophageal was 7.7 cm and that in the stomach was 2.3 cm) (Table 2). Patients in all studies were followed up for more than 2 years.

**Table 1:** The characteristics of patients associated with this study

Author	Country	Study design	Year	Case	M/F	Mean age (range) (years)	Mean symptoms duration (range) (months)	Prior treatment n(%)					Mean follow-up (range) (months)
								EBD	BTI	LHM	Others	Overall	
Hu et al. [12]	China	Prospective	2015	32	17/15	43.6 (18.0~72.0)	40.8 (1.2~600.0)	14 (43.8)	3 (9.4)	3 (9.4)	3 (9.4)	23 (71.9)	30.0 (24.0~44.0)
Peng et al. [13]	China	Retrospective	2017	13	8/5	37.5 ± 13.0	46.8 ± 33.6	3 (23.1)	0 (0)	0 (0)	3 (23.1)	6 (46.2)	46.2 ± 4.1
Zhang et al. [14]	China	Retrospective	2017	32	16/16	43.3 (16.0~79.0)	24.0 (2.4~336.0)	0 (0)	1 (3.1)	0 (0)	0 (0)	1 (3.1)	27.0 (24.0~51.0)
Chen et al. [15]	China	Prospective	2015	27	11/16	13.8 (6.0~17.0)	20.4 (2.4~36.0)	5 (18.5)	1 (3.7)	0 (0)	9 (33.3)	15 (55.6)	24.6 (15.0~38.0)
Lv et al. [16]	China	Retrospective	2016	6	1/5	49.0 (21.0~72.0)	96.0 (24.0~300.0)	0 (0)	0 (0)	0 (0)	2 (33.3)	2 (33.3)	34.5 (median)
Zhang et al. [17]	China	Retrospective	2017	21	9/12	38.0 (15.0~64.0)	26.0 (10.0~360.0)	0 (0)	3 (14.3)	0 (0)	1 (4.8)	4 (19.1)	42.0 (9.0~62.0)
Meng et al. [18]	China	Retrospective	2017	32	13/19	44.8 ± 11.6	24.0 (12.0~60.0)	0 (0)	0 (0)	0 (0)	0 (0)	0 (0)	25.0 ± 11.0
Duan et al. [19]	China	Retrospective	2017	123	63/60	42.1 (14.0~74.0)	60.0 (6.0~396.0)	11	4	0 (0)	3	18	30.0 (24.0~46.0)
Teitelbaum et al. [20]	USA	Prospective	2017	36	16/20	55.0 (20.0~88.0)	NR	NR	NR	0 (0)	18 (50.0)	18 (50.0)	65.0 (median)
Amy Tyberg et al. [21]	USA	Prospective	2017	51	24/27	54.2	134.4	0 (0)	0 (0)	43 (84.0)	NR	45 (88.0)	24.4 (12.0~52.0)
Total				373	178/195	42.1	52.5	33 (8.8)	12 (3.2)	46 (12.3)	39 (10.5)	132 (35.4)	30.0

*NR* Not reported, *M/F* Male/Female, *EBD* Endoscopic balloon dilatation, BTI:Botulinum toxin injections, LHM:Laparoscopic Heller myotomy

**Table 2:** The treatment characteristics of the peroral endoscopic myotomy(POEM)

Author	Mean submucosal tunnel length (cm)	Mean Myotomy length (cm)			Mean Operative Time(range)(min)	Mean hospital stay (days)	Complications n(%)							Replacement therapy n(%)
		Esoph-ageal	Gastric	Overall			Subcutaneous emphysema	Pneumo-thorax	Mucosa perforation	Mediastinal emphysema	Pneumope-ritoneum	Others	Overall	
Hu et al. [12]	NR	8.0 (5~11)	2.3 (2~5)	10.3 (7~14)	63.7 (22~130)	3.9 (1.0~29)	4 (14.3)	1 (3.6)	12 (37.5)	10 (35.7)	7 (25.0)	NR	28 (87.5)	0 (0)
Peng et al. [13]	11.0	NR	NR	7.5 ± 1.3	93.4 ± 23.5	4.0 (3.5~4.5)	0 (0)	1 (7.7)	0 (0)	0 (0)	0 (0)	0 (0)	1 (7.7)	0 (0)
Zhang et al. [14]	9.3 (4.0~14.0)	NR	NR	8.2 (3.0~15.0)	34.9 (17.9~88.6)	NR	0 (0)	1 (3.1)	0 (0)	0 (0)	4 (12.5)	1 (3.1)	6 (18.8)	0 (0)
Chen et al. [15]	NR	7.4 (5~9)	2.2 (2~4)	9.6 ± 1.1 (7~11)	39.4 ± 17.4 (21.0~90.0)	3.2 ± 1.2 (1.0~7.0)	7 (36.8)	3 (15.8)	5 (19.2)	10 (52.6)	9 (47.4)	NR	9 (34.6)	1 (3.7)
Lv et al. [16]	12.0 (median)	NR	NR	10.0 (median)	76.7 ± 22.3	6.0 (median)	0 (0)	0 (0)	1 (16.7)	NR	NR	NR	6 (100)	0 (0)
Zhang et al. [17]	11.7 (7.0~18.0)	NR	NR	5.6 (3.0~10.0)	58.9 (20.0~141.0)	5.0 (5.0~7.0)	0 (0)	1 (4.8)	0 (0)	1 (4.8)	1 (4.8)	0 (0)	3 (14.3)	0 (0)
Meng et al. [18]	13.0 (12.0~14.0)	NR	NR	8.0 (7.0~8.0)	72.5 (40.0~180.0)	8.0 (6.0~10.0)	4 (12.5)	0 (0)	0 (0)	0 (0)	0 (0)	0 (0)	4 (12.5)	0 (0)
Duan et al. [19]	13.6 ± 1.2	NR	NR	10.5 ± 1.3	60.3 ± 10.3	5.5	NR	NR	3 (2.4)	NR	NR	11 (8.9)	14 (11.4)	0 (0)
Teitelbaum et al. [20]	NR	NR	NR	NR	130.0 (60.0~220.0)	1.0 (1.0~2.0)	0 (0)	0 (0)	0 (0)	0 (0)	0 (0)	1 (2.8)	1 (2.8)	0 (0)
Amy Tyberg et al. [21]	NR	NR	NR	13.5 (12~15)	NR	NR	0 (0)	0 (0)	6 (NR)	0 (0)	0 (0)	2 (NR)	7 (13.0)	0 (0)
Total	11.72	7.7	2.3	9.3	66.7	4.2	15 (18.99)	7 (8.86)	27 (34.2)	21 (26.6)	21 (26.6)	15 (18.99)	79 (21.2)	1 (0.3)

### Clinical outcome

The mean follow-up period was 30.0 months. The mean preoperative and postoperative Eckhart scores decreased from 7.4 to 1.4, respectively. Mean postoperative LESP significantly decreased from 32.8 mmHg prior to treatment to 13.7 mmHg (Table 3). During the average follow-up period, the total success rate was 92.9% ± 6.1%. The clinical success rate of POEM was 100% in 26 children [15] and 83% in 19 elderly patients [20].

**Table 3:** The postoperative outcomes

Author	Mean LESP (mmHg)		Mean Eckardt score		GER /RE n(%)	Clinical success n(%)
	Preoperative	Postoperative	Preoperative	Postoperative		
Hu et al. [12]	37.9 (21.9~70.3)	12.9 (7.7~22.5)	7.8 (4.0~12.0)	1.4 (0~5.0)	8 (25.8)	30 (96.8)
Peng et al. [13]	NR	NR	7.5 ± 1.3	2.6 ± 1.5	1 (8.3)	9 (83.3)
Zhang et al. [14]	39.2 (19.4~78.0)	19.0 (10.8~30.6)	7.2 (4.0~11.0)	1.4 (0~5.0)	6 (18.8)	29 (90.6)
Chen et al. [15]	31.6 ± 9.1 (16.0~45.3)	12.9 ± 4.3 (6.1~21.0)	8.3 ± 1.6 (6.0~12.0)	0.7 ± 0.8 (0~2.0)	5 (19.2)	26 (100)
Lv et al. [16]	35.3 ± 4.4	12.8 ± 2.7	7.0 (median)	1.5 (median)	1 (16.7)	NR
Zhang et al. [17]	31.9 (21.9~67.1)	20.3 (6.0~41.0)	5.0 (4.0~10.0)	1.0 (0~4.0)	3 (14.3)	20 (95.2)
Meng et al. [18]	28.0 ± 10.9	12.6 ± 6.5	6.94 ± 2.14	1.1 ± 1.0	6 (18.8)	NR (94.8)
Duan et al. [19]	34.5 ± 7.06	11.8 ± 5.1	7.3 ± 1.8	0.56 ± 0.78	6 (4.9)	121 (98.4)
Teitelbaum et al. [20]	23.0 ± 15.0	NR	6.2 ± 2.6	1.7 ± 1.6	2 (13.0)	19 (83.0)
Amy Tyberg et al. [21]	NR	NR	7.98 ± 2.11	1.72 ± 1.5	0 (0)	48 (94.0)
Total	32.8	13.7	7.4	1.4	38 (10.2)	302 (92.9)

*LESP* Lower esophageal sphincter pressure, *Eckardt’s* Eckardt score, *GER /RE* Gastroesophageal reflux/ Reflux esophagitis

Clinical success: Eckardt score≤3

### Complications

In total, 79 (21.2%) patients had complications. Fifteen (19.0%) patients had subcutaneous emphysema, 7 (8.86%) had pneumothorax, 27 (34.2%) had small mucosal perforation, 21 (26.6%) had mediastinal emphysema, and 21 (26.6%) had pneumoperitoneum. In addition, 15 (18.99%) patients developed other complications or adverse events. All complications were successfully managed during procedure. During the follow-up period, there were 38 cases of GER/RE. The mean incidence rate was 13.9% ± 7.7%. No patients passed away due to POEM.

## Discussion

The treatment of achalasia has advanced over the decades, expanding from drug therapies to include modalities such as endoscopic balloon dilation, botulinum toxin injection, and LHM. The treatment of achalasia has gradually transformed from invasive to minimally invasive. The goals of therapy include decreasing the Eckardt score, reducing LESP, improving esophageal emptying, and relieving symptoms [23–26]. With the advancement of endoscopic devices and techniques, a novel minimal invasive treatment approach, POEM has been developed for treating achalasia [7–9, 27]. Presently, several researchers have reported the effectiveness and safety of POEM. von Renteln et al. reported that the sustained clinical success rate was 82.4% in patients with follow-up period of > 1 year [22]. The efficiency of POEM was reported to be 82–100% by the White Paper Committee et al. in 2014 [30]. Inoue et al. reported the long-term effective rate of 88.5% in 2015 [31]. Youn et al., in their study with follow-up period of > 1 year, reported a range of efficiency of 82.4–100% [10]. However, no prior reviews or meta-analyses have reported the efficacy of POEM with long-term follow-up. Different from the previous systematic reviews, we explored the long-term outcome (median or mean follow-up period of > 2 years) of POEM for achalasia. In our review, the overall mean follow-up period was 30.0 months. Based on our analysis, we found a high overall clinical success rate of 92.9% for POEM, which was similar to the results of previous studies (e.g., White Paper Committee [30] and Youn [10]).

Before the introduction of POEM, LHM was considered an effective method for the treatment of achalasia [1, 28, 29]. During a short follow-up period, some studies have reported that POEM had similar efficacy and safety as LHM [13, 32, 38]. In the study by Peng et al. [13], no significant difference was observed in the myotomy length between POEM and LHM. Improvement in the quality of life after POEM was similar to that after LHM. They demonstrate that the myotomy length was similar between LHM and POEM. However, compared with LHM, POEM may has several obvious advantages, including (i) shorter operation duration; (ii) shorter length of hospital stay; (iii) lower incidence of postoperative complications and higher success rate; (iv) milder postoperative pain; and (v) lower rates of GER and RE [13, 32–38].

Our study indicated that the overall average length of myotomy was 9.3 cm. In recent years, it has become a hot topic about whether long or short muscle incision should be used to treat achalasia. It has been reported that these two therapies had similar success rate and complication rate. However, the operation time of short myotomy was less obviously [39, 40]. In the future, a large number of conclusive studies are needed to assess the long-term outcomes of the short myotomy group versus long myotomy group.

Currently, a circular myotomy (CM) is a frequently used in POEM procedure. Li et al. and Duan et al. have reported a comparison between circular and full-thickness myotomy (FTM). In their studies, the average procedure duration was shorter for FTM than for CM [19, 41]. The study by Li et al. demonstrated that the short-term efficacy and safety were similar between FTM and CM [41]. Duan et al. reported that FTM might increase the incidence of GERD or RE [19]. So, further studies are needed to evaluate the long-term results of FTM versus CM.

Although a few studies reported that the complication rate was 0–30% [1, 6, 42, 43], severe complications of POEM have not been reported. The study by Crespin et al. indicated that the incidence of GERD or RE was 0–19% [1]. Our review demonstrated that the overall incidence of complications was 21.2% and that of GERD/RE was 10.2% during the 2-years’ follow-up. All these adverse events can be alleviated by conservative treatment.

Our review has some limitations. The results of our summary and analysis might have a selection bias because most of our analysis samples came from Asia and from a single endoscopy center. The sample size of the analysis was also relatively small. Large sample and multi-center studies are needed to clarify the effectiveness and safety of POEM.

## Conclusions

Our systematic review examined 10 studies about POEM for achalasia. We found that POEM is effective and safe for the treatment of achalasia during the 2 years’ long-term follow-up duration. Further randomized controlled trial comparing POEM with other treatment modalities with large sample size are wrranted in future.

## Abbreviations

BTI: botulinum toxin injections
CM: circular myotomy
EBD: endoscopic balloon dilatation
FTM: full-thickness myotomy
GER: gastroesophageal reflux
LESP: lower esophageal sphincter pressure
LHM: laparoscopic Heller myotomy
POEM: Peroral endoscopic myotomy
RE: reflux esophagitis
